# Implementation of Measurement-Based Care in Mental Health Service Settings for Youth: A Systematic Review

**DOI:** 10.1007/s10567-024-00498-z

**Published:** 2024-10-30

**Authors:** Emma D. Whitmyre, Christianne Esposito-Smythers, Roberto López, Debora G. Goldberg, Freda Liu, Annamarie B. Defayette

**Affiliations:** 1 Psychology Department, Children’s National Hospital, 111 Michigan Avenue NW, Washington, D.C. 20010 USA; 2 Psychology Department, George Mason University, Fairfax, VA 22030 USA; 3 Department of Psychiatry and Biobehavioral Sciences, University of California, Los Angeles, Los Angeles, CA 90095 USA; 4 School of Medicine in Psychiatry and Behavioral Sciences, University of Washington, Seattle, WA 98195 USA; 5 Department of Psychiatry, University of Rochester Medical Center, Rochester, NY 14642 USA

**Keywords:** Measurement-based care, Youth, Implementation, Barriers

## Abstract

Measurement-Based Care (MBC) is the systematic use of patient-reported data to inform care decisions and monitor treatment progress. MBC has been shown to improve patient outcomes across medical and mental health treatment settings for adults and youth. While many studies have examined the use of MBC in specific care settings, few have focused on the implementation of MBC among youth populations or across care settings. While a review has shown that use of MBC benefits youth, no published reviews exist that summarize the successful strategies and barriers to implementation models across studies in youth service settings. To address these gaps, the present systematic review (*N* = 25 studies) focuses on the implementation of MBC across four youth service settings, including outpatient mental health centers, medical centers/pediatric clinics, schools, and clinical psychology training clinics. Results suggest that few studies employ consistent implementation models or strategies to guide efforts. Further, there is significant overlap in the successful strategies employed as well as the barriers to implementation of MBC across youth service settings, at the client, clinician, and organizational levels. Broadly, the authors recommend on the basis of findings that future implementation work in youth service settings: incorporate comprehensive training in the use of MBC for clinicians; incorporate stakeholder feedback into the implementation process for initial and sustained use; employ digital measurement feedback systems to deliver MBC that allow for real-time feedback and continuous technical support; and employ a health equity lens in implementation efforts to help address disparities in access to and use of MBC so that all youth and families may benefit from this evidence-based practice.

## Introduction

Measurement-Based Care (MBC), also referred to as routine outcome monitoring, patient-reported outcome measures, and progress monitoring, is the systematic use of patient-reported data to inform care decisions (Lewis et al., 2020; Scott & Lewis, 2015). Previous research has established MBC as an evidence-based practice (APA Presidential Task Force on Evidence-Based Practice, 2006; Bickman et al., 2011; Jensen-Doss & Hawley, 2010; Hogan 2003; Sapyta et al., 2005) and is used to both improve service delivery more broadly as well as to monitor patient outcomes. MBC can be delivered electronically, such as through integration into existing Electronic Health Records (EHRs) or via standalone Measurement Feedback Systems (MFSs).

As MBC has been shown to improve patient outcomes across medical and mental health treatment settings (Lewis et al., 2020), accrediting bodies, payers, and behavioral health professional organizations have pushed for the broad adoption of MBC. For example, in 2018, the Joint Commission on Accreditation of Healthcare Organizations, which has approximately 21,000 healthcare organizations under its purview, significantly strengthened its standard for MBC in behavioral health settings (CTS.03.01.09). It now requires that organizations: (1) use a *standardized* tool or instrument to monitor each client’s progress in achieving care, treatment, or service goals; (2) analyze the data generated through standardized monitoring of each client to inform care, treatment, and services; and (3) aggregate and analyze data gathered through standardized monitoring for the population seen to evaluate organization-level outcomes derived from care, treatment, and service provision (Black et al., 2020). Given these mandates, use of MBC, particularly through MFSs and within EHRs, will undoubtedly increase in popularity and adoption. The American Psychological Association is also pushing for a MBC professional practice guideline to improve clinical service delivery, and implementation of MBC- and evidence-based treatments (Boswell et al., 2023).

However, most service settings do not use evidence-based measures to systematically monitor treatment outcomes for their patients nor are they required in mental health services, which may contribute to the inconsistent effectiveness of evidence-based practices for youth (Hoagwood et al., 2001). Specifically, while not the sole factor, research suggests that failure to implement MBC across mental health settings might contribute to the discrepancy between the promising results from randomized controlled trials and the poor outcomes commonly seen in real-world clinical practice settings (Weisz et al., 2017).

Over three decades of research – focused primarily on adult populations – has illustrated that use of MBC can improve treatment outcomes (Parikh et al., 2020, Fortney et al., 2017). However, the evidence for using MBC in youth service settings is less robust and more disparate (i.e., only 6 RCTs currently exist in the literature). Methods across these trials are reported inconsistently due to the variability of study participants (e.g., youth self-report, caregivers, teachers), study design, and implementation strategies. Despite these inconsistencies, studies demonstrate that use of MBC benefits youth (Parikh et al., 2020), and youth who receive services that incorporate MBC into clinical care, relative to those who do not, experience a faster reduction in their mental health symptoms (Bickman et al., 2011; Warren et al., 2018; Wolpert et al., 2012). Effect sizes of MBC in youth psychotherapy are especially large when clinicians have access to the client’s data (Bickman et al., 2011). Notably, the clinical utility of MBC for youth has the potential to help improve patient-provider communication, patient engagement, clinician treatment fidelity, and per the adult literature, costs of care (Delgadillo et al., 2017; Janse et al., 2017). The use of MBC can also strengthen quality improvement efforts (Chaney et al., 2011) by allowing for monitoring of patient outcome data while undergoing systematic changes in clinical operations. Therefore, it is important to learn how to best implement MBC across different youth service settings for effective use.

### General Issues Around Implementing MBC

Despite the known efficacy of MBC and the push to implement it in a manner that allows for consistent and sustainable use, there remain several barriers to effective implementation. Broadly, multilevel barriers to implementation of MBC in youth and adult behavioral healthcare have been well-documented (Lewis et al., 2020). Specifically, prior research has demonstrated that lack of training in the use of MBC (Levine et al., 2017) is a significant barrier. Thus, clinicians do not regularly or systematically engage in use of MBC when it is available (Jensen-Doss et al., 2020; Hall et al., 2014a, 2014b) and overly rely on their own clinical judgment (Walfish et al., 2012). Clinicians also worry about the impact of MBC on the therapeutic alliance (Norman et al., 2014). Additionally, some clinicians feel nervous about the potential use of unfavorable client feedback data (i.e., lack of client symptom improvement) for performance evaluation and find it challenging to make the time to administer or organize measures (Boswell et al., 2015; Jensen-Doss & Hawley, 2010; Wolpert et al., 2014). Finally, integration of the use of technology to deliver MBC in mental health service settings, such as via MFSs, presents specific challenges, such as the need for additional resources to cover the costs of clinician training, usage, and support (Bickman et al., 2000).

Specific challenges to implementation of MBC in youth mental health service also exist. For example, caregivers might feel that use of MBC adds to the burden of completing forms in service settings which can lead to the inconsistent use (Hall et al., 2013 & 2014b). Yet, integration of caregiver report of mental health symptoms is an important consideration when selecting measures to incorporate into MBC across settings that serve youth. A recent national study suggests that up to 77% of 1,500 mental health service providers exclusively use assessments of youth functioning that are not empirically validated or administered in a standardized manner (Cook et al., 2017) and lack caregiver reports. Despite these existent issues, it is worth noting that there is very limited research in this area given that few studies are published on experiences of use of both youth and caregiver support with MBC use. While there is some evidence that clinicians worry about the potential negative impact of collecting additional measures (e.g., caregiver report) on rapport building with clients, they do not always find this to be the case in actual clinical practice (Connors et al., 2021; Cuperfain et al., 2021). Alternatively and importantly, individualized measures developed by clinicians for their clients are a clinically valid approach to using MBC in addition to use of standardized measures (Jensen-Doss & Hawley, 2010).

Moreover, developmental considerations of measures, such as caregiver reporting and developmental needs in assessment, may affect the implementation models and strategies selected to guide implementation efforts in youth settings, that is, when the models and strategies are used. Despite their utility, implementation models and strategies are rarely used consistently in MBC implementation work either (Lewis et al., 2020), particularly in youth service settings (Bruns et al., 2008). Results from a national survey of youth-serving outpatient mental health clinics demonstrated that less than a third of these facilities implement MBC at all (Cook et al., 2017). Thus, there is much work to be done in this area.

Previous systematic reviews and meta-analyses conducted on MBC across service settings have primarily focused on exploring client symptom improvement (Elmquist et al., 2010; Gondek et al., 2016; Krägeloh et al., 2015; Lambert et al., 2003; Shimokawa et al., 2010; Tam & Ronan, 2017; Waldrop et al., 2017; Williams et al., 2016; Zimmerman & McGlinchey, 2008), with 24% of clients in community mental health settings demonstrating a significant increase in symptoms over the course of treatment, as opposed to 14% of clients in the managed care setting (Warren et al., 2018). However, little attention is given to implementation processes associated with effective and sustained use of MBC. To our knowledge, a review focused on the implementation of MBC in youth mental health service settings has not been published. The inconsistencies in studies focused on use of MBC in youth service settings as well as the low uptake of MBC in these settings could be due to the differences in implementation processes of MBC and nuances between studies (e.g., implementation strategies used, informants utilizing MBC). Thus, the objectives of this systematic review are to (1) review models and strategies used to implement MBC in youth-serving settings; (2) summarize the barriers and use of facilitators to implementation of MBC in these settings; and (3) offer recommendations for future work in this area. This review includes an examination of the primary settings where youth receive mental health care and where the majority of findings related to MBC use have been reported in the literature, including community-based outpatient mental health centers (CMHCs), medical centers/hospital-based pediatric clinics/units, schools, and clinical psychology training clinics.

## Methods

### Search Strategy and Study Eligibility Criteria

A systematic review of the literature was conducted in a manner consistent with the Preferred Reporting Items for Systematic Reviews and Meta Analyses (PRISMA) statement (Moher et al., 2009) to identify peer-reviewed studies on the implementation of MBC in youth service settings, by two independent reviewers. Searches of electronic databases (PsychInfo, Medline, Google Scholar) were conducted using specific keywords (measurement-based care or measurement feedback system or routine outcome monitoring or MBC, mental health, and children or adolescents or youth or child or teenager), for articles published between 1995 and May 2024. Search strategies were the same across databases.

Included studies met the following criteria: (1) examined the implementation of MBC in mental health services for youth; (2) focused exclusively on settings that provide mental health services, such as community-based care and pediatric medical centers; and (3) identified strategies or barriers associated with implementation processes in study procedures and findings. The reference lists from included papers and other systematic reviews (Elmquist et al., 2010; Lewis et al., 2020) were also reviewed for papers that matched inclusion criteria. Exclusion criteria included studies that (1) were not in English; (2) not implementation focused; (3) focused on use of MBC for non-mental health-related issues or teaching purposes; (4) did not focus on the use or system of the MBC (e.g., focused on creation of a measure in a MFS that delivers MBC); (5) used the acronym “MBC” to refer to something else; and (6) examined adult-focused settings and did not include youth (ages 0–18). Exclusion criteria for the Medline search also included articles that were duplicates from the PsychInfo search. A search of Google Scholar and review of reference lists in the aforementioned papers produced 8 additional papers that met the inclusion criteria and were not included in the PsychInfo or Medline searches. Coding of the papers was conducted a priori such that each study was examined for descriptive information (e.g., setting, population/age, youth sample size), mode of delivery (e.g., MFS, paper–pencil), measures employed, implementation model/framework and strategies used, and barriers and facilitators to implementation discussed. When multiple articles were drawn from the same study (e.g., Bickman et al., 2016 and Gleacher et al., 2016), they were counted as a single study in our review.

## Results

**Fig. 1:**
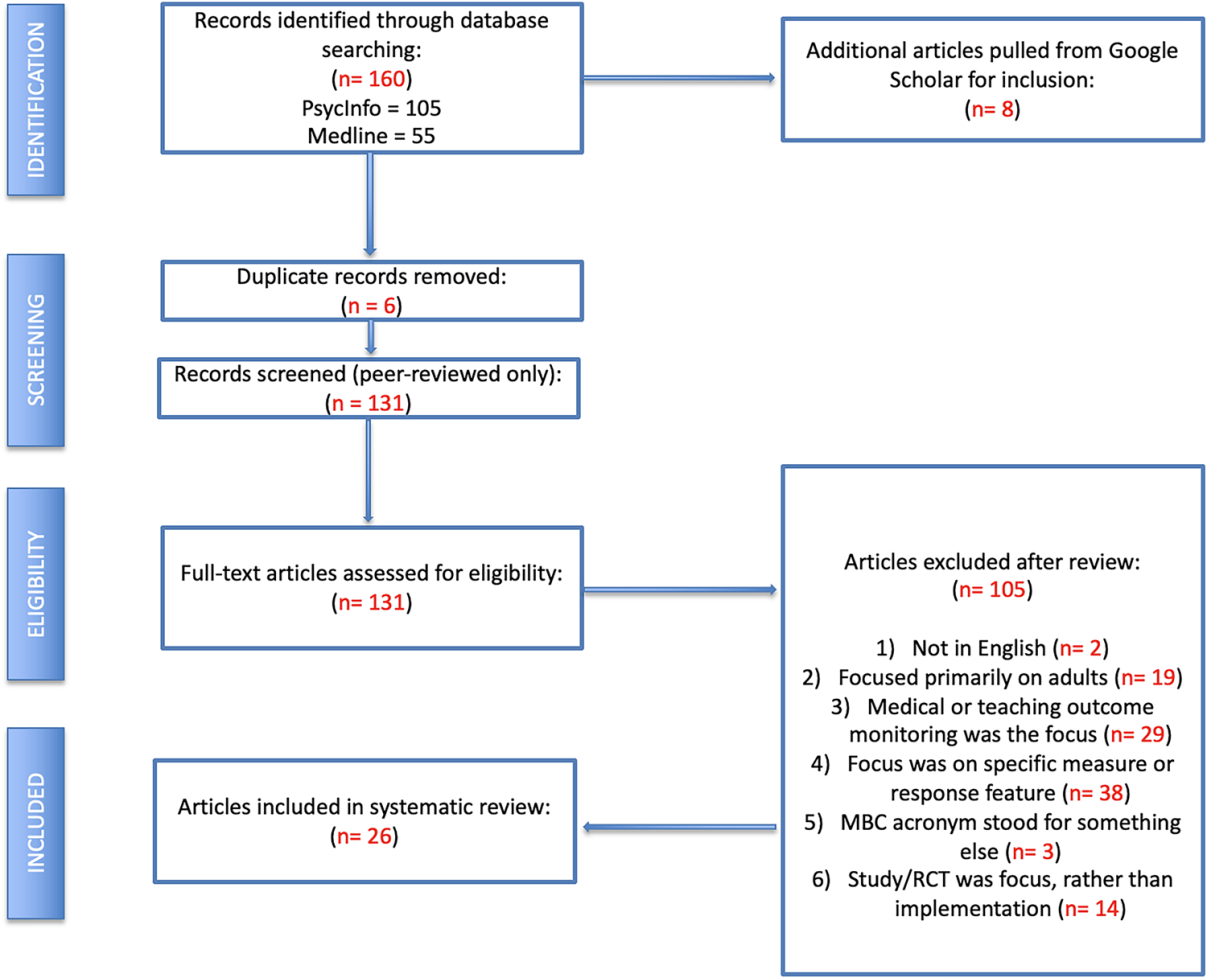
Inclusion and exclusion of articles in systematic review

The initial search utilizing the aforementioned keywords identified 131 peer-reviewed articles (see Fig. 1). Twenty-six articles, representing data from 25 studies, met full inclusion criteria. Table 1 includes all of the articles that met the inclusion criteria along with a description of coding. Of the final set of articles included that examined implementation of MBC, 17 were focused on CMHCs, 5 were in medical centers/hospital-based pediatric clinics/units, 1 was in a single school, and 3 were in clinical psychology training clinics. Given that so few studies were conducted in settings outside of CMHCs, results are largely combined across settings.

**Table 1:** Descriptions of Implementation Models/Strategies and Results from Articles Included in Systematic Review

Author, Year	Setting	Population/Age	Youth Sample Size	Vehicle For Delivery of MBC	Symptoms Assessed by MBC	Symptom Measures in System	Implementation Model/Framework, Type of Observation, and Outcomes	Implementation Strategies Used	Barriers/Facilitators
Bantjes et al., 2018	Clinical Psychology Training Clinic	Adolescents, 14–18 (some early adulthood)	No report	Small system- 2 measures used—PP	General functioning and distress	Outcome Rating Scale and Kessler Psychological Distress Scale	Model: Not reported Client-level determinants: client report/researcher observation (case studies) Outcomes: noted that measure tracking had utility; however, significant problems with implementation of MBC	Heavy emphasis on training	**Barriers**: supervisors believed it would negatively impact therapeutic process, poor client understanding of measures. **Facilitators**: carefully making changes to routine practice, use of research evidence to validate importance of MBC during training
Batty et al., 2013	Community Outpatient Tx	Children and Adolescents, age not reported	127	CAMHS (Outcome Research Consortium in the UK)—WB	Broad mental health	HoNOSCA, Strengths and Difficulties Questionnaire (SDQ) (parent, teacher, youth), Conners’ rating scale (teacher, parent), Children’s Global Assessment Scale (C-GAS)	Model: Not reported Client-level determinants: clinical report Outcomes: principle of MBC supported by stakeholders; however, barriers to implementation led to low-outcome measurement completion (completion for < 1/5 cases)	Training and involvement of key stakeholders in implementation planning and trainings, HIT strategies	**Barriers**: lack of training and resources, clinicians’ perceived limitations in measures, lack of regular assessment feedback. **Facilitators**: support of a large healthcare system, integration with EHR, administrative resources
Bickman et al., 2016	Community Outpatient Tx	Children and Adolescents, 11–18	257	Contextualized Feedback Systems (CFSTM; Bickman et al., 2011, 2012)—WB and some PP (hand entered into system)	Internalizing and externalizing symptom severity, and therapeutic alliance, life satisfaction, motivation for treatment, hope, treatment expectations, caregiver strain, and service satisfaction	Peabody Treatment Progress Battery and The Symptoms and Functioning Severity Scale (SFSS: Bickman et al. 2010)	Conceptual framework provided by Powell and colleagues (2012) to describe different implementation strategies emerging from two sites Client-level determinants: client report/clinical report Outcomes: the clinic utilizing feedback demonstrated an enhanced outcome for implementation of MBC	Consultation services, collaborative workgroups to help clinicians prepare to integrate MFS into their workflow and post-implementation coaching, HIT strategies	**Barriers**: paper versions of measures forms down feedback process, need for changes to MFS, low staff confidence in MFS **Facilitators**: ongoing leadership support and consultation, increased senior leadership involvement for staff engagement, in-house staff support, flexible completion of MBC measures for families (e.g., allowing families to complete measures before their first appointment)
Black et al., 2020	Community Outpatient Tx	Children and Adolescents, age not reported	No report	MFS (OWL Outcomes)—WB	Broad mental health, mania, trauma, insomnia, suicidal ideation and behavior, non-suicidal self-injury, eating, alcohol and substance misuse/abuse, psychosis, mania	Digital library of selected evidence-based behavioral health measures for youth (see Appendix; Black et al., 2020)	Model: CFIR model Client-level determinants: clinical report/researcher observation Outcomes: strong uptake of MBC through implementation process, through HIT methods	HIT strategies	**Barriers**: lack of resources (time, leadership, capital) **Facilitators**: theory-driven and evidence-based pre-implementation assessment to create implementation plan, staged implementation to enhance trial ability and make changes, leadership engagement, clear communication, mandated use of MBC, training for staff
Bruns et al., 2018	Community Outpatient Tx (one large agency and one regional mental health center)	Children and Adolescents, 5–18	42	EHR-online software system developed through a partnership between a university research team and a small behavioral health-focused software developer—WB	Broad mental health and PTSD	Standardized outcome measures (not reported) and the Client Satisfaction Questionnaire	Model: Not reported Client-level determinants: client report/clinical report Outcomes: use of EHR systems can promote the use of MBC; however, minimal evidence of impact on service quality, fidelity, and client satisfaction	HIT strategies and training in use of online EHR software package	**Barriers**: functionality issues that compromised ease of use, lack of training in use of /purpose of MBC in EHR (control group) **Facilitators**: MBC integrated into EHR, key stakeholder guided sites in using MBC, training (experimental group)
Childs et al., 2022	Intensive Outpatient Program (IOP), Hospital Based	Adolescents, 12–18	371	EPIC optimization team built measures into EHR for administration, scoring and tracking through MyChart Patient Portal	Anxiety and depression	PROMIS pediatric anxiety and pediatric depression	Model: Consolidated Framework for Implementation Research (CFIR) Client-level determinants: clinical report/researcher observation Outcomes: aggregate completion rates of measures were strong for both programs, across all four stages	Five Domains from CFIR model addressed in model developed pre-implementation, with ongoing quality improvement work and assessments with families, collection of feedback from providers, HIT methods	**Barriers:** perceived disruption to clinical care, “voltage drop” perceived by leadership, burden of measure interpretation **Facilitators**: administrative/complexity support for families completing measures, education/consultations with providers, leadership engagement, building self-efficacy of providers, trainings
Cooper et al., 2019	Clinical Psychology Training Clinic	Children, age not reported	85	MFS (OWL Outcomes)—WB	Broad functioning and symptom-specific measures (not specified)	Not reported	Model: Fixsen, Naoom, Blasé, Friedman, and Wallace (2005) process framework Client-level determinants: client report/clinical report Outcomes: clients with positive attitudes toward MBC use and education around MBC use were more likely to complete measures over time (significant rate of change across implementation phase)	Pre-implementation interviews and assessment of client and clinician reported attitudes toward use of MBC to leverage intraorganizational factors (i.e., to address potential barriers to implementation), HIT methods	**Barriers**: time burden, perceived disruption to therapeutic process, lack of previous staff training in MBC **Facilitators**: regular and early training in use of/evidence base for MBC
Gatto et al., 2024	Community-Based Psychology Training Clinic	Children and Adolescents, 1–17	79	OWL (Families provided online account to complete PROMs)	Broad mental health/based on symptoms presentation and diagnostic interview	Patient-Rated Outcome Measures (PROMs-see article for comprehensive list of measures-229 MBC measures available in the system)	Model: Standardized Clinic Outcome Research & Evaluation Project Client-level determinants: client report (family feedback about MBC use)/clinical report (completion rates) Outcomes: feedback from families and results demonstrate successful use of MBC and utility of implementing MBC in a training clinic	Utilized strategies from previous implementation work (Cooper et al., 2021), with focus on identifying barriers/facilitators to MBC implementation, and a focus on attitudes toward MBC and utilization, HIT methods	**Barriers**: limited resources for implementation process and for trainings, time burden of multiple informant reporting, lack of focus on youth-specific issues within MBC implementation during training **Facilitators**: positive attitudes toward MBC use by families, clinicians’ view of the usefulness of MBC, training (added later in implementation process due to resource issues initially)
Gleacher et al., 2016	Community Outpatient Tx	Children and Adolescents, 11–18	No report	Contextualized Feedback System—WB	Broad mental health, therapeutic alliance, life satisfaction, motivation for treatment, caregiver strain	Peabody Treatment Progress Battery	Model: Aarons et al., 2011 framework Client-level determinants: researcher observation through interviews with clinicians Outcomes: clinicians perceived more barriers than facilitators to implementation of MBC; despite reporting high levels of organizational and leadership support	Semi-structured interviews with clinicians to examine multilevel factors that influence the uptake and implementation of an MFS that delivers MBC to address barriers and leverage facilitators to MBC implementation, HIT strategies	**Barriers**: Clinician difficulty using MBC technology (i.e., MFS), late arrival of families to sessions, technological issues for families, functionality issues with MFS, administrative burden **Facilitators**: commitment to staff engagement and leadership supports, individual characteristics of clients (e.g., more session time for measure completion), high relative priority for clinicians, training support
Kotte et al., 2016	Community Outpatient Tx	Children and Adolescents, age not reported	No report	MFS—WB	Problem Severity, Functioning (severity and frequency of symptoms across common youth- and parent-reported types of problems), Satisfaction with Services, Hopefulness, “Restrictiveness of Living Environments”	The Ohio Scales	Model: EPIS Model Client-level determinants: researcher observation of interviews with care coordinators Outcomes: researchers observed emergence of facilitators and barriers that are in line with implementation framework used	Post-implementation interviews with care coordinators to assess their perceptions around the administration of MBC and use of client data to aid in clinical decision-making, HIT methods	**Barriers**: reliability and validity of measures and clinician perception of measures, lack of administrative supports, inconsistency of use/discussion in meetings, lack of family motivation/compliance difficulty, inadequate explanation of system to caregivers **Facilitators**: clinician recognition clinical utility of MBC, compliance with organizational demand, and positive attitudes toward MBC
Lamers et al., 2015	Medical- Residential Tx (semi-residential psychiatry)	Children, 6–12	46	Patient- Reported Outcome Measurement Information System (ProMISe)—WB	Broad mental health, parenting stress, family engagement	SDQ, Health of the Nation Outcome Scales, Working Alliance Inventory, Parenting Stress Questionnaire, Family Engagement Questionnaire (Dutch versions)	Model: Not reported Client-level determinants: clinical report Outcomes: initial treatment factors and measure completion played a role in overall completion of measures, and implementation of MBC, across time	Enhancing administrative support (e.g., time, staffing, technological troubleshooting) to facilitate implementation efforts and transitioning to use of paper and electronic questionnaires prior to implementation, HIT strategies	**Barriers**: time pressure, poor case manager opinion of MFS, and usability of questionnaires, youth co-morbidity, single parenthood. **Facilitators**: administrative support (including email reminders), use of paper *and* electronic questionnaires
Lavik et al., 2018	Community Outpatient Tx	Adolescents, 12–19	22	Not reported	Internalizing and externalizing disorders	Not reported	Model: Not reported Client-level determinants: clinical report/researcher observation Outcomes: findings emphasize that MBC implementation outcomes should be understood in the context of developmental phases of clients, and goals/values of clients	Pre-implementation semi-structured interviews to engage / make any necessary changes to implementation protocol	**Barriers**: poor adolescent perception of measures and buy-in to using MBC, poor clinician attitudes toward MBC, MBC not a relative priority **Facilitators**: engaging adolescents in MBC, sensitivity of measures to developmental phases, focus on adolescent’s goals and values in measure selection
Liu et al., 2019	Medical-child and adolescent psychiatry department (regional pediatric tertiary-care center)	Children and Adolescents, 6–18	No report	MFS (cloud-based, Health Insurance Portability and Accountability Act–compliant software)—WB	Broad mental health (not specified)	Digital library of 40 evidence-based behavioral health measures for youth	Model: Not reported Client-level determinants: clinical report Outcomes: initial and repeated measure completion rates in MBC increased during implementation. Clients with public insurance were half as likely to receive MFS account as those privately insured	Quality improvement (QI) project that leveraged Health Information Technology to implement MBC, HIT strategies	**Barriers**: insurance disparities, lag between real-time feedback and use of MFS for MBC, clinicians acting as “gatekeepers” to assist families with measure completion **Facilitators**: None reported
Lui et al., 2021	Community Outpatient Tx (Los Angeles County Department of Mental Health [LACDMH] service system)	Children and Adolescents, age not reported	No report	Not reported	Broad mental health	Not reported	Model: EPIS Model Client-level determinants: researcher observation Outcomes: additional supports for MBC implementation efforts (e.g., administrative and leadership) assist with completion/use	Pre- & post-implementation assessment of clinician demographics & reported attitudes toward MBC	**Barriers:** None reported **Facilitators**: Sensitivity to language and interpretability of measures, automation of MBC process, dedicated support/administrative staff to help with MBC process, mandating MBC
Lyon et al., 2019	School	Children and Adolescents (middle and high school), age not reported	No report	MFS (Mental Health Integrated Tracking System)—WB	Broad mental health	Patient Health Questionnaire–9 (PHQ-9); Generalized Anxiety Disorder–7; SDQ	Model: Not reported Client-level determinants: clinical report/researcher observation Outcomes: clinicians in the MFS condition (i.e., digital MFS access, consultation) demonstrated increase in MBC practice/use and attitudes/skill, while those in the control group did not change significantly	HIT strategies and consultation support in one condition of a RCT to compare implementation effectiveness/ sustainment. Training based on strategies in literature (e.g., interactive didactic presentations, clinicians’ personal reflections on their current assessment practices, specific practice activities, and small group discussions critical to uptake of new skills	**Barriers**: lack of time for consultation/ support, negative clinician attitudes toward usefulness of measures. **Facilitators**: support and consultation calls, pre- and ongoing training efforts, adaptation of MFS for school setting
Monga et al., 2023	Medical-across pediatric hospital setting	Children and Adolescents	No report	Plans to trial Voxel (external, patient-friendly platform)-project is abstract currently	Focus on anxiety and depression	PROMs	Model: Not reported Client-level determinants: not reported Outcomes: implementation model set for next steps based on focus group feedback	Environmental-scan and focus groups (n = 57) used to evaluate need for MBC and set implementation framework, desire to use HIT methods	**Barriers:** need for provider and patient training/education around MBC, managing sensitive data in systems **Facilitators**(for building implementation model): **s**takeholders engaged in the process were physician leaders, clinical operational directors, clinical staff, Youth and Family Advisory Panel members across hospital departments, measures that are brief and limit time burden
Moran et al., 2012	Community Outpatient Tx	Children and Adolescents, up to age 18	No report	CAMHS (Outcome Research Consortium in the UK)—WB	Broad mental health (predominant focus on ADHD and ASD)	SDQ, C-GAS	Model: Not reported Client-level determinants: client report/clinical report Outcomes: implementation processes/focus groups noted that it is important to include service users throughout stages of implementation of MBC	Focus groups with caregivers and youth (service users) to examine quality of life measures and guide implementation	**Barriers**: confusing language on measures, purpose of measures difficult to understand for families, need for multiple measures, fears around service access if data shows child improvement in treatment **Facilitators**: brief measures that are simple to complete and understand
Norman et al., 2014	Community Outpatient Tx (2 clinics)	Children and Adolescents, under age 18	No report	Children and Young People Improving Access to Psychological Therapies (CYI IAPT) program, measures entered into electronic system	Broad mental health and service experience	Strengths and Difficulties Questionnaire (SDQ), the Revised Children’s Anxiety and Depression Scale (RCADS) and the Experience of Service Questionnaire (Chi ESQ)	Model: Not reported Client-level determinants: N/A Outcomes: clinician attitudes [number of advantages (55%) only slightly outweighing that of the number of disadvantages (45%)] used to shape future implementation process	Use of Child and Adolescent Mental Health Services Outcome Research Consortium (CORC) (2005), a learning collaboration of practitioners, managers and academics exploring MBC, focus on attitudes toward use	**Barriers:** clinician concern about administrative burden and clinical utility (e.g., measures unrepresentative of population), administrative burden, depersonalization of clinical work **Facilitators**: positive attitudes regarding use, including clinical utility of symptom monitoring and ability to fine tune trainings needed, perception of adding utility to clinical workflow and meeting goals
Purbeck et al., 2020	Community Outpatient Tx (and 1 residential facility)	Children and Adolescents, ages not reported	No report	The Clinical Improvement through Measurement Initiative (CIMI)—WB	Functional impairment, trauma history (e.g., onset, duration, and frequency), emotional/behavioral problems	The Child Behavioral Checklist, SDQ, UCLA Posttraumatic Stress Disorder Reaction Index for the DSM-V, among others (authors only specified measures listed above)	Model: Heuristic proposed by Proctor et al. (2011); CFIR model Client-level determinants: researcher observation of clinician/staff feedback Outcomes: clinicians and staff agreed that implementation process and technology were acceptable, feasible, and appropriate, and were willing to use MBC to guide case conceptualization, and other factors were noted to enhance adoption of MBC (see barriers)	Combination of externally led training and consultation components as suggested by Harding et al. (2011); all teams met monthly with study staff via WebEx for 13 months to direct and refine implementation	**Barriers:** clinician difficulty using MBC technology, time burden, lack of measure availability for informant reporting **Facilitators**: support of external change agents (implantation purveyors) and formally appointed internal implementation leaders and CIMI “champions”
Sale et al., 2021	Community Outpatient Tx	Children and Adolescents, age not reported	229	MFS (OQ Analyst, Lambert et al., 2010)—WB	General functioning and distress	Youth Outcome Questionnaire-30 (Y-OQ)	Model: Not reported Client-level determinants: client report/clinical report Outcomes: client symptoms decreased faster for those routinely using MFS per caregiver report; however, fidelity to MFS use was dependent on trainee status (e.g., lower fidelity for trainees vs. clinicians)	Front desk staff were instructed to administer MFS measures; training delivered by the MFS developers 3 months prior to the start date, HIT methods	**Barriers:** negative clinician attitudes toward MBC **Facilitators**: training by MFS developers
Sichel & Connors, 2022	Community Outpatient Tx (4 Centers)	Children and Adolescents, age not reported	No report	MFS-private label of ACORN developed with funding from the state’s Accountable Care Organ-ization	Internalizing concerns, externalizing concerns, and working alliance, with child- and parent-reported versions	“Client Feedback Form”	Model: None reported Client-level determinants: clinical report related to measure use by clinicians Outcomes: clinicians in the higher MBC use group reported more facilitators to implementation (and more positive attitudes toward implementation) than clinicians in the low MBC use group. Need for individual-level implementation strategies to target clinician needs, skills, and perceptions was highlighted	Implementation organized into three phases: 1) clinician groups identified based on clinician-level characteristics; 2) Qualitative analyses of clinician data conducted to understand multilevel barriers and facilitators to MFS implementation; 3) reflection/analysis of clinician-level variables to inform future implementation model, HIT methods	**Barriers:** need for individual-level strategies to target clinician knowledge and self-efficacy, and clinician attitudes/perceptions **Facilitators**: clarity of system, appropriateness, and feasibility of the MFS and its measures; clinician knowledge and skills; client preferences and behaviors; and incentives and resources (e.g., CE credits, time back)
Trivedi et al., 2019	Medical—Primary Care	Adolescents, 12 -18	No report	EHR (VitalSign6) – WB	Depression	PHQ-9	Model: The RE-AIM Model Client-level determinants: N/A Outcomes: model implemented as part of quality improvement project and to be further measured within RE-AIM framework	HIT methods	**Barriers**: low rates of attendance at appointments in primary care **Facilitators**: MBC integrated into EHR, conceptualization of mental health as chronic health condition, and electronic medical applications
Van Sonsbeek et al., 2021	Community Outpatient Tx	Children and Adolescents, 4—17	432	ROM-system (NetQ-ROM) - WB	Broad mental health, quality of life, and satisfaction with treatment	SDQ, KIDSCREEN (quality of life), and satisfaction with treatment scale (only at end of tx)	Model: Not reported Client-level determinants: client report/clinical report Outcomes: following implementation, plan to further measure client-level factors (symptom severity) and session-level factors (rates of dropout)	HIT methods	**Barriers**: None reported **Facilitators**: specific and concrete feedback from clinician to family, discussions about measures and patient results during case meetings or consultation, improvements in youth mental health
Victor et al., 2023	Community Outpatient Tx- specialty clinic for suicidal youth (outpatient and IOP)	Children and Adolescents, age not reported	No report	Paper measures-results securely transmitted to an encrypted database, where assessments are scored and compiled with previously collected data -transitioning to electronic system w/pandemic	Broad mental health	SMFQ, SCARED, CALS, PSQI, CRAFFT, ARI, ASQ	Model: Not reported Client-level determinants: clinical report related to measure use by clinicians Outcomes: results suggest that MBC is feasible and acceptable for use with suicidal youth (84% of expected measures completed)	Integration with other longer-term implementation procedures at STAR Center (www.starcenter.pitt.edu/about), current study focused on clinician usage of and attitudes toward MBC	**Barriers:** appropriateness of measures (developmental and otherwise), patient/time burden, sensitivity of measures to change over time, clinician failing to share measure or client refusal to complete **Facilitators**: clinician buy-in and attitudes, leadership engagement, timely and regular feedback to clients and clinicians
Waschbusch et al., 2020	Community Outpatient Tx	Children and Adolescents, age not reported	No report	Penn State Psychiatry Clinical Assessment and Rating Evaluation System for Youth (PCARES Youth)—WB	Broad mental health, ASD, affective reactivity, prosocial emotions/behaviors, caregiver strain	Digital library of 16 selected evidence-based behavioral health measures for youth (see Table 1; Waschbusch et al., 2020)	Model: Not reported Client-level determinants: researcher observation of clinician/stakeholder report Outcomes: clinicians and stakeholder interviews and feedback noted support for MBC implementation, with suggestions for improving implementation model (e.g., including feedback in MBC system for clinicians during implementation/integration into EHR)	Conducted surveys with clinicians, youth, and stakeholders to assess opinions about measures included in MBC and the system used. Used these data to guide decisions around implementation	**Barriers**: assessments in system deemed to be too long or included redundant questions, lack of graphs with measures, lack of integration with medical record, clinicians receiving strong (positive or negative) feedback from caregivers, administrative burden **Facilitators**: stakeholder support and encouragement, willingness of caregivers to participate in MBC prior to first visit, clinician understanding and familiarity with measures and score interpretation
Woodard et al., 2023	Community Outpatient Tx	Adolescents, ages 12–18	56	MFS- OQ System	Broad mental health and therapy alliance	Youth Outcome Questionnaire	Model: Not reported Client-level determinants: clinical report/researcher observation Outcomes: greater consultation dosage (more time) significantly predicted a higher implementation index for MBC (ß = 0.27, SE = 0.06, p < .001), and more consultation predicted higher fidelity using MBC	Ongoing consultation calls with clinicians and fidelity monitoring, following methodology from the preferred reporting guidelines for observational studies (STROBE) within the Community Study of Outcome Monitoring for Emotional Disorders in Teens (COMET), HIT methods	**Barriers:** low call attendance or less time discussing cases during consultation **Facilitators:** ongoing consultation calls (1–2 per week, focused on use of MBC/incorporation into practice, troubleshooting technical issues), spending more time discussing cases during consultation

*WB* web based, *PP* paper and pen, “Broad Mental Health” includes symptom measures for depression, anxiety, attention, and disruptive behaviors

### Implementation Models and Strategies

Overall, nine out of the 26 articles included mention of implementation models. Studies included in this review paper mentioned use of the Consolidated Framework for Implementation Research (CFIR; Damschroder et al., 2009), Aarons and colleagues (2011) conceptual framework, the Exploration, Preparation, Implementation, Sustainment (EPIS) model (Aarons et al., 2011), and Powell and colleagues (2012) conceptual framework to guide implementation efforts. Additionally, the Reach, Efficacy, Adoption, Implementation and Maintenance framework (RE-AIM) (Glasgow et al., 2001) was used to guide the implementation efforts in a primary care setting study, and the Fixsen, Naoom, Blasé, Friedman, and Wallace (2005) process framework was used in the context of a clinical psychology training clinic.

In contrast to implementation models, multiple implementation strategies were described in the studies reviewed. General strategies common across all service settings included use of training which varied in scope across studies (Bantjes et al., 2018, Batty et al., 2013; Bruns et al., 2008; Childs et al., 2022; Cooper et al., 2019; Gatto et al., 2024; Lavik et al., 2018; Lyon et al., 2015; Norman et al., 2014; Trivedi et al., 2019) and use of technology to collect, analyze, and link patient data through digital systems, also known as Health Information Technology (HIT) strategies (Bantjes et al., 2018; Black et al., 2020; Bruns et al., 2008; Childs et al., 2022; Cooper et al., 2019; Gatto et al., 2024; Gleacher et al., 2016; Lyon et al., 2015; Monga et al., 2023; Moran et al., 2012; Sichel & Connors, 2022; Trivedi et al., 2019; Van Sonsbeek et al., 2021; Waschbusch et al., 2020; Woodard et al., 2023). In many of the studies reviewed, HIT strategies, such as a MFS or EHR, were used to deliver MBC. Of note, studies also used various forms of web-based platforms, paper forms, and electronic routine outcome monitoring systems with results entered into clinical records routinely. Additionally, some systems included integration of not only clinical measures, but also scoring and tracking of these measures in the patient portal (e.g., through MyChart) (e.g., Childs et al., 2022). Another strategy mentioned in literature from CMHCs and clinical psychology training clinics was the involvement of key stakeholders, such as clinicians, caregivers, and/or youth, in implementation planning (Batty et al., 2013; Bruns et al., 2008; Lavik et al., 2018; Monga et al., 2023; Victor et al., 2023). For example, focus groups, semi-structured interviews, or pre-implementation surveys were used to obtain feedback in areas such as measure selection and mode of measure administration as well as general barriers and facilitators to implementation of MBC within a MFS (Bantjes et al., 2018; Cooper et al., 2019; Gleacher et al., 2016; Moran et al., 2012; Waschbusch et al., 2020).

### Barriers and Facilitators to Implementation

All barriers and facilitators or the individual characteristics and contextual factors that enhanced or impeded implementation processes, including those that are less common across settings, are discussed below. They are subdivided into clinician-, client-, and organizational-level strategies.

### Barriers

#### Clinician-Level Barriers

With regard to clinician-level barriers in general, dissatisfaction with MBC measures, negative attitudes around use of MBC, and lack of/inadequate training appeared most frequently across articles as factors negatively impacting implementation. Importantly, dissatisfaction with measure selection in MBC use within the school setting is highly specific to youth settings, as elaborated below.

#### Dissatisfaction with MBC Measures

Specifically, across CMHCs, medical center, and school settings, clinicians perceived that there were limitations to the measures included in some MBC systems and the resultant clinical validity of the data (Batty et al., 2013; Kotte et al., 2016; Lavik et al., 2018; Lamers et al., 2015; Lyon et al., 2019; Victor et al., 2023). For example, the relevance of the measures to the clients served was a concern noted in CMHC and medical centers (Batty et al., 2013; Lamers et al., 2015; Norman et al., 2014). Relatedly, a lack of parent- and teacher report versions of measures, which prevented comparison across informants (Purbeck et al., 2020), as well as lack of concordance of measures with clients’ treatment goals, values, or attitudes (Lavik et al., 2018), were cited as concerns in CMHCs. Within a school mental health clinic, hesitancy about the reliability and validity of the selected MBC measures was associated with lower use of MBC (Lyon et al., 2015).

#### Negative Attitudes Around MBC Use

Another clinician-level barrier examined in studies, common to medical center, community mental health, and clinical psychology training clinics, was a negative attitude toward MBC use (Bantjes et al., 2018; Childs et al., 2022; Cooper et al., 2019; Lamers et al., 2015; Norman et al., 2014; Siche & Connors, 2022). This negative attitude may be driven, in part, by a lack of previous training in MBC in graduate work (Cooper et al., 2019) as well as the belief that MBC is disruptive to the therapeutic process in settings where evidence-based practices are not fully embraced. For example, in one training clinic, clinical supervisors expressed concern about the potential negative impact of MBC use on the therapeutic process between student therapists and their clients as well as the clinician training process in general (Bantjes et al., 2018).

#### Lack of Training in MBC

A few additional clinician-level barriers were also cited in the studies reviewed. Within a CMHC, this included a lack of any training or specific training on MBC measures for youth and their clinical utility (Batty et al., 2013; Bruns et al., 2008; Gatto et al., 2024; Monga et al., 2023), difficulty using the MFS technology that delivers MBC (Gleacher et al., 2016; Liu et al., 2019; Purbeck et al., 2020), and inconsistent review of client MBC measures by clinicians in team meetings (Kotte et al., 2016; Batty et al., 2013). Dissatisfaction with certain features of the MBC system employed was also a barrier to consistent use in medical center settings. These features included a “lag time” between real-time measurement feedback (i.e., unable to see measure results/graphs instantaneously with measure completion during sessions) and clinician feedback delivery (i.e., unable to share associated graphs with clients during session) (Lieu et al., 2019). One additional barrier, encompassed in the context of a clinical psychology training clinic, was the time burden associated with the use of MBC (Cooper et al., 2019).

#### Client-Level Barriers

Common client-level barriers to successful use of MBC broadly included system usability issues, poor client engagement, and lack of measures in clients’ native language/client English language proficiency, which appeared across articles most frequently as factors negatively impacting implementation.

##### System Usability Issues

Across CMHCs and clinical psychology training clinics, difficulty with the use of the technology that supports MBC was cited as a client-level barrier (Bantjes et al., 2018; Gleacher et al., 2016; Kotte et al., 2016; Waschbusch et al., 2020). This difficulty may be prompted, in part, by lack of computer literacy (Gleacher et al., 2016; Waschbusch et al., 2020) as well as burdensome technical requirements and complicated system designs in general (Bruns et al., 2008; Gleacher et al., 2016) and perceived time burden of multiple informant reporting (Gatto et al., 2024).

#### Poor Client Engagement

Another common client-level barrier cited within CMHCs and medical centers is poor understanding of the clinical utility of MBC and how these data are used. With CMHCs, this barrier was associated with low motivation to complete MBC measures and annoyance with repeated measures (Kotte et al., 2016). Highly specific to the youth settings reviewed, caregiver-perceived irrelevance of youth measures and concerns around being discharged from care if measures indicated improvement in child’s symptoms (Moran et al., 2012; Waschbusch et al., 2020) were important barriers uncovered in this review. This barrier may be associated with poor explanation of MBC by clinicians (Kotte et al., 2016) and lack of systems that employ real-time feedback (Liu et al., 2019).

A number of factors related to the broader care context that may impact MBC, or the system employed to deliver it, were also cited as client-level barriers in CMHCs and medical settings. Within medical settings, this includes familial factors associated with limited parental time and heightened stress, such as greater youth mental health co-morbidity, one sole youth caregiver, and higher education level/more demanding jobs (Lamers et al., 2015). Relatedly, low patient attendance rates at visits (Trivedi et al., 2019), particularly among families covered by Medicaid (vs. privately insured) (Liu et al., 2019), were noted as a barriers to completion of MBC measures. Within CMHCs, late arrival of families to sessions (Gleacher et al., 2016; Waschbusch et al., 2020) as well as environmental distractors for those asked to complete measures outside of the therapy session (Kotte et al., 20^2016^16) were cited as barriers to MBC measure completion. This is an important consideration for youth service settings, given that parents oftentimes report on their young child’s behalf or work to remind older children to complete their measures on time.

#### Lack of MBC Resources in Multiple Languages

A final client-level barrier associated with MBC use is related to client language and reading literacy. Within CMHCs, a lack of measures in caregivers’ native language was cited as a barrier to measure completion (Moran et al., 2012; Waschbusch et al., 2020). Within clinical psychology training clinics, poor caregiver comprehension of questions on MBC measures was cited as a barrier (Bantjes et al., 2018).

##### Organizational-Level Barriers

The organizational-level barriers negatively impacting implementation, and most frequently cited across service settings, include the time burden associated with MBC administration and the resources that it requires. This is similar to the organizational-level barriers encountered in adult service settings.

#### Time Burden

Across CMHC, medical center, and school settings, the administrative time burden associated with tasks such as the administration of measures used for MBC and addressing technical problems within the electronic systems on behalf of clinicians and/or clients were cited as barriers (Batty et al., 2013, Black et al., 2020; Gleacher et al., 2016; Kotte et al., 2016; Lamers et al., 2015; Liu et al., 2019; Lyon et al., 2015; Victor et al., 2023; Waschbusch et al., 2020). Relatedly, a barrier common to CMHCs and schools is a lack of resources (e.g., monetary, support staff) to readily implement MBC systems with sufficient levels of consultation and support (Batty et al., 2013, Black et al., 2020; Lyon et al., 2015).

#### Resources Required

Additionally, within CMHCs, low leadership support, poor networking between developers and adopters (Batty et al., 2013, Black et al., 2020; Gatto et al., 2024; Kotte et al., 2016; Waschbusch et al., 2020), and lack of integration of MBC with client medical records and clinical workflow (Waschbusch et al., 2020) complicated implementation efforts. Finally, completion of measures with pen and paper by caregivers slowed the use of MBC and further limited timely feedback to clinicians given the added administrative burden of entering the form into the electronic system (Bickman et al., 2016).

### Facilitators

#### Clinician-Level Strategies

Many common clinician-level strategies to implementation of MBC were cited which generally include clinician and staff engagement, comprehensive training, and post-training consultation. Across CMHC, medical, and clinical psychology training clinics, importance of clinician and staff buy-in, including positive attitudes toward MBC use, were most frequently highlighted as facilitators (Cooper et al., 2019; Kotte et al., 2016; Liu et al., 2019).

#### Comprehensive Training

A number of facilitators may enhance clinician buy-in, such as high-quality training and post-implementation support. Indeed, across all four service settings, structured and systematic training was cited as successful strategy for MBC implementation efforts (Black et al., 2020; Bruns et al., 2018; Cooper et al., 2019; Lyon et al., 2019). Within CMHCs, this training was used to enhance clinician knowledge and self-efficacy in use of MBC and decrease the time burden associated with MBC related administrative tasks (Black et al., 2020; Bruns et al., 2008). Inclusion of consultation services and collaborative workgroups in training were particularly helpful in facilitating clinician preparation efforts to integrate MFS into their workflow (Bickman et al., 2016; Purbeck et al., 2020; Sale et al., 2021; Van Sonsbeek et al., 2021), in particular while working with both caregivers and clients (i.e., completing twice as many measures per client in the system) in youth settings.

#### Post-Training Consultation

Studies conducted within CMHCs and school settings also highlighted the importance of post-training consultation to implementation efforts (Bickman et al., 2016; Bruns et al., 2008; Childs et al., 2022; Gleacher et al., 2016; Lyon et al., 2015; Woodard et al., 2023). For example, within school settings and highly specific to youth, continuous follow-up consultation post-training, such as support and consultation calls provided by expert consultants around the use of the MFS with youth, served as successful strategies for implementation (Lyon et al., 2015). It was also noted that the use of “online message boards” can be used to augment the delivery of support services for MBC in school settings (Stirman et al., 2013; Lyon et al., 2015), along with timely and regular feedback on system use to clinicians (Victor et al., 2023). Of note, a great amount of time spent on consultation calls predicted a higher implementation index for MBC in community mental health (Woodard et al., 2023).

#### Technological Aids

Additional facilitators to clinician-level implementation efforts were also noted. Within medical centers and clinical psychology training clinics, clinician buy-in was enhanced when certain technological features were present in the MBC systems that were adopted. This includes the direct integration of the MFS used to deliver MBC into the EHR systems (e.g., SMART; Substitutable Medical Apps and Reusable Technology) and use of web-based MBC interfaces to coordinate electronic record keeping and symptom tracking between various health care systems or offices within medical settings (Trivedi et al., 2019). Use of brief measures in the context of a MFS that include auto-scoring and graphing has enhanced implementation efforts in clinical psychology training clinics (Cooper et al., 2019). Indeed, regular use of MFSs/EHRs to deliver MBC increases the frequency and amount of data collected from families, which in turn, offers clinicians more information about client progress and a greater understanding of its utility (Norman et al., 2014; Trivedi et al., 2019). Regular use of MFSs by clinicians also improves the sustainability of MBC practices in the school settings (Lyon et al., 2015). Within CMHCs, the inclusion of a formally appointed MBC implementation leader or Panel (Monga et al., 2023; Purbeck et al., 2020) as well as general clinician willingness to address administrative barriers and comply with organizational demands (Childs et al., 2022; Kotte et al., 2016) were found to be helpful. Use of measures in the MBC system that were developmentally sensitive and aligned with client goals and values were also found to facilitate clinician use of MBC (Lavik, 2018). Finally, within a clinical psychology training clinic, reviewing results of client MBC measures in clinical supervision meetings enhanced clinician use and uptake of MBC (Cooper et al., 2019).

#### Client-Level Strategies

Generally, most frequently cited client-level strategies for successful MBC implementation included favorable measure selection and understanding of MBC as well as client assistance. Notably, measure selection and client assistance are particularly important for consideration in youth service settings given developmental variability among clients and use of caregiver/teacher report measures in addition to client measures.

#### Measure Selection

Specifically, within both CMHCs and clinical psychology training clinics, use of measures that were perceived to be developmentally sensitive and applicable to youth and family needs and goals were described as facilitators (Cooper et al., 2019; Lavik et al., 2018; Monga et al., 2023). Within CMHCs, a positive perception of MBC was further facilitated by psychoeducation about MBC provided by clinicians (Cooper et al., 2019), consistent use of MBC over the course of care, and clinician sharing of assessment results with families and associated treatment implications (Van Sonsbeek et al., 2021).

#### Client Assistance

Another common facilitator across CMHC and medical settings centered around the provision of client assistance and options associated with measure completion. For example, within a CMHC, offering simple and brief verbal instructions when families had trouble understanding written directions was perceived to be helpful (Moran et al., 2012). Administrative email reminders sent to caregivers (with clinicians copied) to complete measures as well as allowing caregivers to select between electronic or paper MBC measures was related to higher completion rates medical settings (Lamers et al., 2015). Relatedly, broad administrative support and allowing families to complete measures within several days before their first appointment was identified as a facilitator in CMHCs (Bickman et al., 2016; Childs et al., 2022; Waschbusch et al., 2020).

##### Organizational-Level Strategies

Common successful strategies at the organizational level included integration of MBC into existing initiatives, systems, and structures, as well as access to resources to support MBC implementation, as similarly noted across the adult literature on MBC implementation.

#### Integration of MBC into Existing Initiatives

With regard to the integration of MBC into existing initiatives/systems, integration of MBC into larger-quality improvement efforts and standard clinical care (Black et al., 2020; Lui et al., 2021) in CMHCs, clinical/staff workflows (Black et al., 2020; Lyon et al., 2015) in CMHCs and schools settings, and EHRs (Black et al., 2020; Lamers et al., 2015; Lui et al., 202l; Trivedi et al., 2019) in CMHC and medical settings, all served as organizational-level strategies.

#### Resources to Support Implementation

Similarly, across CMHC and medical settings, having sufficient financial and administrative resources facilitated organizational-level success (Batty et al., 2013; Lamers et al., 2015; Sichel & Connors, 2022; Trivedi et al., 2019). In one CMHC, fundraising was conducted to gain monetary support for the MFS used to deliver MBC and associated consultation (Black et al., 2020). Other successful organizational-level strategies within CMHCs included support from leadership and stakeholders, the development and clear communication of goals and staff incentives for using MBC, and a commitment to staff engagement (Bickman et al., 2016; Black et al., 2020; Bruns et al., 2008; Childs et al., 2022; Gleacher et al., 2016; Lui et al., 2021; Victor et al., 2023; Waschbusch et al., 2020).

## Discussion

The present systematic review focused on the implementation of MBC in youth service settings. Our review revealed a significant amount of overlap in barriers to MBC implementation, as well as facilitators to use of MBC across the settings reviewed. Based on common themes identified in this review, three concrete recommendations are offered to address barriers and facilitate the implementation of MBC into clinical practice across settings with youth. These recommendations are discussed in the context of all four treatment settings, given the overlap in many of the barriers and facilitators across service settings and the fact that few papers exist in some of the settings examined. Of note, the results of our review as indicated above were determinants observed and discussed by the authors, and not all findings were empirically derived based on implementation outcome data. Specifically, while several determinants of implementation were observed, others are linked directly to analyses of implementation outcomes as noted in the literature, such as links between clinician attitudes and MBC fidelity. We shed further light on these recommendations, using supporting evidence, as outlined below.

### Recommendation 1

First, we recommend incorporating comprehensive training in use of MBC for clinicians who work with youth and families as well as continuous support, as needed, post-training. Specifically, receiving training prior to using MBC, or a MFS that delivers MBC, can improve clinician usage rates of MBC, attitudes toward MBC (Bruns et al., 2008), and knowledge and skill sets with MBC practices (Peterson & Fagan, 2017; Warren & Park, 2018). Ideally, training in evidence-based practices, such as MBC, should be provided at the “earliest stages of professional training” when clinicians are learning new practices. This helps to ensure that clinicians will implement knowledge correctly and understand how to integrate MBC into their clinic workflow (Bantjes et al., 2018; Peterson & Fagan, 2017; Warren & Park, 2018). Embedding training in MBC in the context of graduate education (i.e., training clinics) has been associated with implementation successes (Cooper et al., 2019) and sets the stage for continuation of this practice in future service settings.

Our review also suggests that there are important topics to integrate into training with clinicians to address potential barriers and enhance facilitators to implementation. First, the training should include information about why MBC is important and how it improves quality of patient care. Time should also be devoted to describing when and how feedback should be delivered to youth and families. This includes a discussion of how regular monitoring of symptoms and feedback can be used to enhance rapport, communication, and collaborative decision-making between families and clinicians. The importance of delivering regular MBC feedback to youth and families (i.e., via graphs, charts) over the course of care for optimal outcomes should also be emphasized. It may also be helpful to convey that the results of assessments are not meant to replace clinical judgment but can be used as another valuable source of information that can be integrated into clinical decision-making. For example, MBC can be used to help guide triage decisions upon entry into care, aid in treatment planning, and inform discharge decisions (Jensen-Doss et al., 2020). In addition, if the data collected will be used for additional purposes (e.g., aggregated for program evaluation and/or to meet accreditation standards), this information should also be conveyed so that clinicians have a full understanding of the use and utility of MBC in their practice setting. Such information may ultimately help improve clinicians’ attitudes toward MBC, which in turn, may enhance implementation outcomes.

Training with clinicians should also incorporate psychoeducation around how to discuss MBC with youth and families. For example, clinicians should learn how to describe the utility of assessment measures to both youth and their families and how MBC can be used to enhance treatment outcomes (e.g., faster reduction in mental health symptoms, shorter time to remission). Information on common barriers to MBC use with families, such as limited time outside of session, lack of understanding of relevance/importance of measures, and poor understanding of technology, as well as how to address these barriers, should also be included in clinician training.

Importantly, training should also provide clinicians with ample time and space to ask questions, offer feedback, discuss concerns, and problem solve through potential obstacles. Needed adjustments should be made to the implementation process based on information obtained. For example, adjustments may need to be made to the clinical workflow or measures added to the MBC system, using feedback from clinicians. Continuous support and consultation should also be provided to clinicians post-training to help sustain and/or enhance use of MBC, and any new feedback gathered during this process may also be used to further improve implementation strategies and efforts.

### Recommendation 2

Second, clinicians play a central role in enhancing youth and parent use of MBC. In particular, it is critical for clinicians to engage clients in use of MBC and address barriers to using MBC with families at the start of care (Chiauzzi, 2020). Encouraging clinicians to address barriers to completing measures with youth and families and problem-solving as needed may enhance “buy-in” for using MBC. Setting goals around MBC use with families may also be helpful in this regard (Bickman et al., 2016; Lamers et al., 2015). Ensuring that families understand the clinical relevance of measures for their children’s treatment and mental health (Lavik et al., 2018) is also important. When possible, selecting measures that are focused on the personal goals and values of the unique youth (Lavik et al., 2018) can enhance engagement in MBC. Providing support for completing measures, especially for families or children with literacy problems, as well as offering assessments in a client’s native language, when at all possible (Moran et al., 2012), will also enhance measure completion. Additionally, some caregivers worry that their family will lose service access if symptom improvement is evidenced via MBC. Families should be assured that they will not lose access to needed services as a function of MBC use (Moran et al., 2012). Given research which suggests that sharing weekly, or regular, assessment results with clients may lead to quicker improvement in youth mental health symptoms (Bickman et al., 2011), feedback should be a continuous process throughout treatment. Feedback that incorporates both quantitative (i.e., symptom inventories) and qualitative (i.e., verbal discussion about results of assessments) assessment in the context of outcome monitoring may be particularly beneficial for clients (Turner, 1998).

### Recommendation 3

Third, we recommend using electronic systems to deliver MBC that allow for real-time feedback (i.e., instantaneously score and show assessment results) and offer continuous consultation/technical support for clinicians and families, when possible. The literature suggests that timely and specific feedback through MFSs that deliver MBC is a critical ingredient for improving patient outcomes (Bickman et al., 2011; Mellor-Clark et al., 2016). Clinicians and organizational leaders use feedback from these MFSs in their treatment planning as well as to monitor implementation outcomes in service settings when it is available (Liu et al., 2019). It is recommended that feedback be: (1) readily accessible to clinicians at both the individual level and the team/service level; (2) presented in graphs or short reports that can be shared with families in sessions as part of monitoring treatment progress; and (3) has the ability to be compared across all assessment timepoints over the course of treatment (Batty et al., 2013; Chiauzzi, 2020). Lastly, given that system usability and technological issues can interfere with implementation processes, service settings that employ consultation or technical support for MFSs benefit greatly (Liu et al., 2019). The amount and type of consultation and technical support needed will vary by treatment setting and the complexity of the MBC employed.

### Future Directions

In addition to the key takeaways gleaned from understanding the barriers and successful strategies to implementation of MBC for youth across service settings, there are several important considerations for scaling up implementation and improving access to MBC for families. Lewis and colleagues (2020) created a 10-point research agenda to improve the implementation of MBC in clinical practice broadly, and several of the strategies most important for youth service settings are highlighted below.

A significant issue impacting the implementation of MBC is the associated cost. While training and administrative costs were cited as a barrier in the studies reviewed, MBC systems themselves can also be costly (e.g., EHRs/MFSs that deliver MBC, use of copyrighted measures). Lewis and colleagues (2020) pointed to a need to “align reimbursement structures” to help cover this cost, especially as many systems become electronic. Upward of 95% of caregivers prefer to complete measures electronically rather than by paper and pencil when offered a choice (Waschbusch et al., 2020). This is an important consideration given that caregiver burden is a significant barrier to consistent MBC use. Billing concerns for assessment services are not unique to mental health facilities. They also exist in integrated healthcare settings where many families access services and early identification of mental health issues among youth is possible (Patient-Centered Medical Home; Croghan & Brown, 2010), particularly when electronic systems are available. There are Current Procedural Terminology (CPT) codes for mental health evaluations that can be used to bill for initial assessment procedures (e.g., 90,791, 90,792) as well as brief emotional-behavioral assessments using standardized instruments (e.g., 96,127), but they do not fully cover the costs of MBC systems, in particular if there are multiple bills entered per family (e.g., siblings, caregiver reports, and teacher reports). Unlike mental health treatment for adults, mental health treatment with youth often incorporates family work, which necessitates a different billing structure. Decreasing the costs, advocating for insurance coverage, and identifying funding streams for electronic systems so that optimal MBC can be implemented, including in economically disadvantaged areas, will be critical for eliminating barriers related to the complexity of reimbursement for MBC and enhancing uptake of MBC.

Additionally, future research should be conducted to examine and/or develop free, brief, developmentally sensitive, and psychometrically sound measures that can be optimally used in the context of MBC systems (Bickman et al., 2016; Lewis et al., 2020; Liu & Adrian, 2019). These measures should span multiple mental health areas to mimic the comorbidity commonly experienced by youth who seek treatment services and include versions for multiple informants (child, caregiver) for optimal assessment, given that several measures might be given to a family at the onset of treatment. Relatedly, unlike work with adults, which often focuses solely on self-reported assessments, it may also be important to train therapists in the clinical utility of discrepant reports from youth and family, which is often the rule rather than the exception (De Los Reyes et al., 2017). Measures should also be validated in multiple languages and be culturally sound to ensure that diverse youth/caregivers have access to MBC across service settings (Liu et al., 2019). Notably, research on implementation of MBC is being conducted worldwide (e.g., the Netherlands, the UK, the USA, and South Africa) and measures in multiple languages are being developed. For example, the Patient-Reported Outcomes Measurement Information System (PROMIS) Measures promoted through the American Psychological Association, are brief, cover multiple mental health areas, are validated in over 90 languages (select measures), and are free to the general public (https://www.healthmeasures.net/index.php?Itemid=992). However, there is a fee for their commercial use in electronic MBC systems, which if waived, may help reduce address inequities in access to MBC. Notably, Beidas et al. (2015) and Becker-Haimes et al. (2020) have compiled free and brief standardized instruments for use in youth mental health settings, which could greatly assist with MBC implementation despite noted difficulties with measure access.

In a similar vein, additional research should be conducted on the use of audio computer-assisted self-interviewing and simple response options to decrease barriers to MBC use for those with low literacy and disabilities (visual impairment, reading disabilities, etc.), and for working with youth with developmental delays. Importantly, a model has recently been developed for examining MBC through an equity specific lens (ASPIRE framework-Gaias et al., 2021), as well as a model to improve engagement with racial minoritized youth (STAY- Connors et al., 2022). Using this model to guide future implementation efforts may help address documented inequities in access to and use of MBC so that all youth and families may benefit from this evidence-based practice.
